# Aggressive *Purpureocillium lilacinum* Kerato-Endophthalmitis in a Diabetic Contact Lens Wearer Culminating in Enucleation: A Case Report and Review of the Literature

**DOI:** 10.3390/jof11110789

**Published:** 2025-11-03

**Authors:** Sara Calendino, Jennifer Kenna, Hetal Patel, Jenny Vereecken, Fatima Almutawah, Jeff Fuller, Sameer Elsayed, Johan Delport, Ruchika Bagga

**Affiliations:** 1 Department of Pathology and Laboratory Medicine, Schulich School of Medicine and Dentistry, Western University, London, ON N6A 3K7, Canada; sara.calendino@lhsc.on.ca (S.C.); jennifer.kenna@lhsc.on.ca (J.K.); hetal.patel@lhsc.on.ca (H.P.); jenny.verecken@lhsc.on.ca (J.V.); fatimah.almutawa@lhsc.on.ca (F.A.); jeff.fuller@lhsc.on.ca (J.F.); sameer.elsayed@lhsc.on.ca (S.E.); johan.delport@lhsc.on.ca (J.D.); 2 London Health Science Centre, London, ON N6A 5W9, Canada; 3 Department of Infectious Diseases, Schulich School of Medicine and Dentistry, Western University, London, ON N6A 3K7, Canada

**Keywords:** *Purpureocilium lilacinum*, endophthalmitis, risk factors, antifungals, management

## Abstract

**Introduction:** *Purpureocillium lilacinum* (formerly *Paecilomyces lilacinus*) is an emerging, saprophytic fungus known to cause severe, treatment-refractory ocular infections. It is notoriously clinically resistant to several common antifungal agents, including amphotericin B. Risk factors for *Purpureocillium lilacinum* (*P. lilacinum)* keratitis include contact lens wear, ocular trauma, and local or systemic immunosuppression. **Case Presentation:** We describe the clinical course of a 70-year-old male with type 2 diabetes mellitus and a history of long-term soft contact lens use who presented with a right corneal ulcer. Despite initial treatment with topical voriconazole, the infection progressed over two months to involve the entire globe, resulting in intractable endophthalmitis. Microbiological analysis of corneal scrapings identified *P. lilacinum*, confirmed by MALDI-TOF mass spectrometry and ITS sequencing. Despite the addition of systemic voriconazole, the patient’s condition deteriorated, leading to a painful blind right eye which ultimately needed enucleation. **Conclusions:** This case highlights the aggressive potential of *P. lilacinum* in a host with multiple risk factors. It underscores the critical need for a high index of suspicion, rapid and accurate mycological diagnosis, and immediate, aggressive management. The therapeutic challenges, including intrinsic and emerging antifungal resistance, often necessitate early surgical intervention to prevent catastrophic outcomes.

## 1. Introduction

*Purpureocillium lilacinum* (previously known as *Paecilomyces lilacinum*) belonging to phylum Ascomycota, class Sordariomycetes, order Hypocreales, family Ophiocordycipitaceae is a ubiquitous, hyaline hyphomycete found in soil and decaying organic matter. In recent years this species has been proven useful as a biocontrol agent against pests and for the promotion of plant growth [1]. While once considered a rare contaminant, it is now recognized as an important opportunistic pathogen in humans [1,2,3], particularly in the context of ocular infections [3]. Ocular infection can manifest as keratitis [3], scleritis, or endophthalmitis [4] and is often associated with significant morbidity, including profound vision loss.

The primary risk factors for *P. lilacinum* keratitis are ocular trauma [4] and soft contact lens wear [3,4,5]. Systemic conditions that impair immunity, such as diabetes mellitus and immunosuppression from underlying hematological malignancy, solid organ cancers, chemotherapy, and corticosteroid use further predispose individuals to severe and invasive disease [6,7]. *P. lilacinum* has a predilection for ocular structures, thought to be due to a thermal tolerance of the fungus, with the optimum temperatures for growth and sporulation ranging from 20–25 °C as well as the unique ability of the fungus to produce hydrolytic enzymes and toxins that disrupt intact cell membranes, including corneal epithelium [8,9]. A significant challenge in managing these infections is the organism’s intrinsic resistance to amphotericin B and echinocandins [10,11], leaving triazoles like voriconazole as the mainstay of therapy [12,13]. However, treatment failures with voriconazole have been reported, linked to poor drug penetration or emerging resistance.

While cases of *P. lilacinum* keratitis in diabetic or contact lens-wearing patients have been documented, reports detailing the progression to endophthalmitis requiring enucleation in a patient with this specific combination of risk factors are scarce in the literature [14,15,16,17]. We present a case report of a 70-year-old diabetic contact lens wearer who suffered this devastating outcome, with a focus on the microbiological diagnosis, therapeutic challenges, and public health context.

## 2. Case Presentation

A 70-year-old Caucasian male with a 15-year history of sub-optimally controlled type 2 diabetes mellitus (most recent HbA1c 8.5%) and daily-wear soft contact lens user for over four decades presented to the ophthalmology clinic with a three-week history of escalating pain, photophobia, blurred vision and discharge from his right eye. He reported no recent trauma but admitted to occasional non-compliance with contact lens hygiene protocols.

On initial examination, there was notable right-sided conjunctival injection, without periorbital swelling or erythema. Visual acuity was 20/40 in the right eye. On fluorescein stain, a large ulcerated area with irregular edges at the 7 O’clock location of the central corneal area was noted. There was a crescent-shaped inferior nasal infiltrate with overlying epithelial defect measuring 5 mm × 2.9 mm. The patient was otherwise systemically well and afebrile at the time.

Corneal scrapings were obtained from the base and edges of the ulcer for microbiological investigations including culture and susceptibility. The patient was advised to avoid contact lens use (contact lens holiday) and one drop of 5% topical vancomycin alternating with one drop off tobramycin drops (14 mg/mL) was administered to the right eye on an hourly basis.

**Figure 1:**
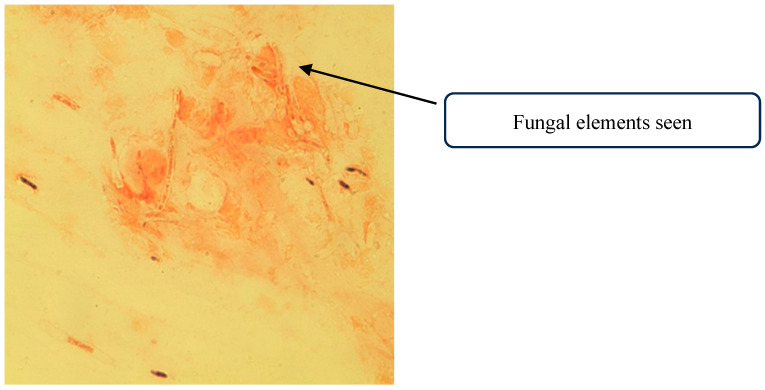
Direct microscopy of the corneal scrapings showing fungal elements.

A corneal scraping was collected, with a portion smeared onto two glass slides for immediate microscopy, while the remainder was inoculated directly onto culture media. A 10% potassium hydroxide (KOH) with Fungi Fluor stain (Polysciences, Warrington, PA, USA) and Gram stain were prepared from the corneal scraping. Several septate hyaline hyphal structures, in keeping with fungal infection were visualized on direct Gram stain (Figure 1).

Blood agar, Chocolate agar and Brain Heart infusion agar were inoculated with the corneal scrapings. All inoculated media showed fungal growth at 30 °C and 35 °C, with visible growth by 48 h and colony maturation within four to six days.

At 48 h, on the obverse rubbery raised greyish colonies were noticed on all the pre inoculated plates, there was no color change noted on the reverse. These colonies were then subcultured onto a Sabouraud dextrose agar with gentamicin (SAB) (Thermofisher Scientific, Waltham, MA, USA) using a sterile wooden needle, embedding the growing fungus slightly into the agar surface. Additionally, at that time a scotch tape mount was performed and hyaline septate hyphae were observed.

**Figure 2:**
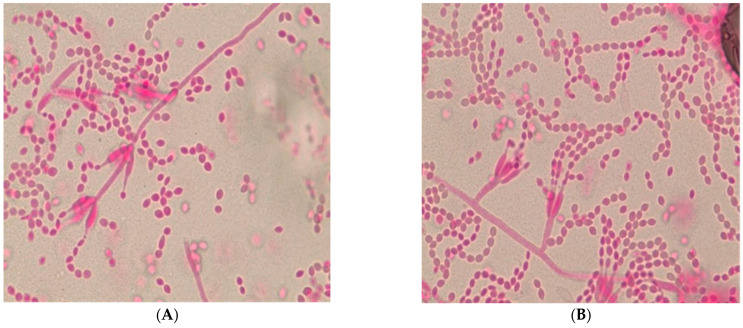
Illustrative Microscopic Morphology of *Purpureocillium lilacinum* (**A**) Lactofuchsin (Sigma, St. Louis, MO, USA) stain (prepared in house using Sigma, USA Acid fuchsin and lactic acid) of a tape mount from culture, demonstrating characteristic phialides with swollen bases and long, tapering necks. (**B**) Chains of oval conidia produced from the phialides.  The microscopy image was taken at 60× magnification.

After two days of incubation on SAB, the fungus was noted to be velvety and raised with a distinct lilac center and a white margin (Figure 2) A second tape mount was performed at the time and showed elongated thin phialides, with small oval conidia being released from the tip of the phialide in delicate chains. The elongated phialides were present both in small groups at the hyphal tip as well as along the edge of the hyphal surface. For rapid identification, an extract from the fungal colony was analyzed using Matrix-Assisted Laser Desorption/Ionization Time-of-Flight Mass Spectrometry (MALDI-TOF MS) with a Bruker Biotyper System and the Filamentous Fungi library (Billerica, MA, USA).The isolate was identified as *P. lilacinum* with a high-confidence score of 2.21.

**Figure 3:**
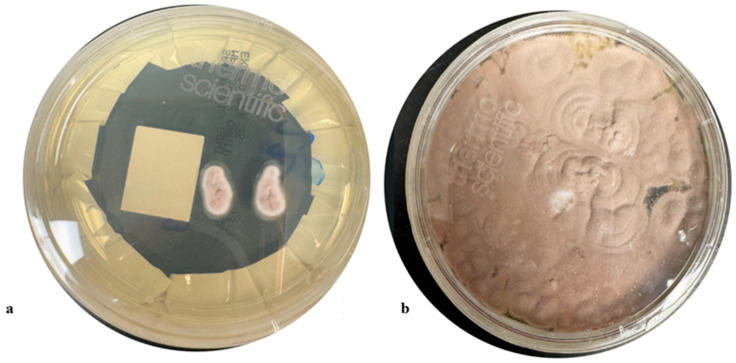
(**a**,**b**) Colony morphology on SAB at 48 h and 120 h, respectively, velvety and raised colony with a distinct lilac center and a white margin.

Based on the distinctive lilac colony morphology, the presence of thin long phialides (Figure 3), and no reduction in growth at 35 °C and MALDI-TOF, identification the fungus was reported as presumptive *P. lilacinum*. To provide definitive confirmation and for epidemiological purposes, the isolate was sent to a public health reference laboratory which *P. lilacinum* using MALDI TOF as well as ITS sequencing. The patient had a temporary tarsorrhaphy of the right eye two weeks later for relief. The patient tolerated the procedure well.

After microbiological identification of *P. lilacinum*, topical tobramycin was discontinued while topical voriconazole (1 mg/mL 1–2 h) was initiated. Over the subsequent two months, the patient’s clinical course was marked by relentless progression. The stromal infiltrate opacified the entire cornea, and signs of scleral and intraocular extension became evident, with the development of vitreous opacities and retinal elevation leading to the possibility of endophthalmitis on B-scan ultrasonography. Oral voriconazole (200 mg twice daily) was added to the topical regimen.

A vitreous tap was done at this time and intravitreal voriconazole, amphotericin B, ceftazidime and vancomycin was given pending culture results to broadly cover for both bacterial and fungal keratitis. The intravitreal tap sample also grew *P. lilacinum*. The diagnosis was again based on the combination of microscopic observations, culture findings, and MALDI-TOF. Culture growth was sent to Public Health Ontario Laboratory for posaconazole and voriconazole susceptibility testing using the Clinical and Laboratory Standards Institute (CLSI) M38-A3 broth microdilution method [18]. Both voriconazole and posaconazole had a minimum inhibitory concentration of 0.5 μg/mL.

**Figure 4:**
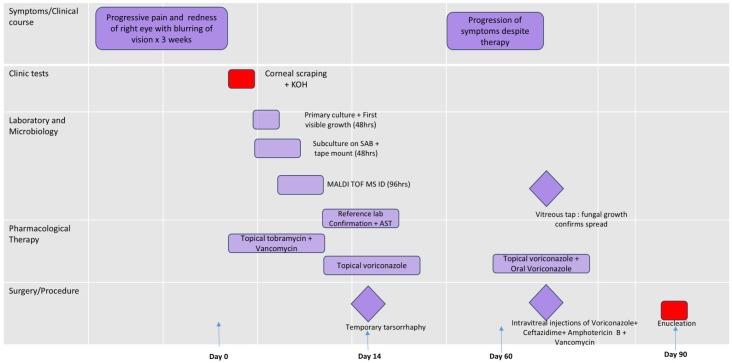
The figure provides a timeline of the case with relevant microbiology and management details.

The patient was therefore continued on both oral and topical voriconazole. Despite this dual therapy, the infection evolved into panophthalmitis, characterized by intractable pain, proptosis, and complete loss of light perception. Given the uncontrolled infection, severe pain, and lack of visual potential, the decision was made to proceed with enucleation of the right eye. (Figure 4 outlines the clinical course of this patient).

## 3. Discussion

*Purpureocillium lilacinum* is an emerging ocular mold that, although infrequently isolated [10], may carry a disproportionate risk of catastrophic visual loss [14,19,20]. Contemporary series estimate its prevalence at 1–4% of culture-positive fungal keratitis, yet it accounts for up to 15% of filamentous fungal keratitis cases ultimately requiring evisceration or enucleation [10,11,12,13,14,15,16,17,21], highlighting its aggressive pathogenic potential. In the present case, the convergence of advanced age, poorly controlled diabetes mellitus, and soft contact lens wear culminated in globe sacrifice despite prompt laboratory confirmation and guideline-based antifungal therapy. Diabetes mellitus compromises neutrophil function, disrupts corneal epithelial integrity, leads to accumulation of advanced glycation end products and creates a hyperglycaemic periocular microenvironment, while contact lenses provide an abiotic surface conducive to biofilm formation and a direct route for fungal inoculation [21]. The interplay of these factors likely accelerated corneal invasion and posterior extension in our patient.

Expedited species-level identification is particularly critical in the *Purpureocillium* genus, as pathogenic potential and antifungal susceptibility vary markedly among species [10,22,23,24] and management differs from other causes of fungal keratitis. Medical management of fungal keratitis relies on intensive topical antifungal therapy. Natamycin 5% remains the first-line agent, and topical voriconazole 1% is an effective alternative, especially for *Aspergillus* and *Candida* species, or in cases unresponsive to natamycin. Systemic antifungals such as oral voriconazole (200 mg twice daily) may be employed for deep stromal involvement or recalcitrant infections. Adjunctive measures, including cycloplegics for symptomatic relief and strict avoidance of corticosteroids during active infection, are essential to optimize outcomes and prevent progression.

Amphotericin B exhibits poor activity against *P. lilacinum* isolates and higher mortality has been noted in patients treated with amphotericin B (10) distinguishing it from *P. variotii*, which generally retains broader antifungal susceptibility and is associated with less severe ocular disease. Consequently, understanding the comparative features of *P. lilacinum* vs. *P. variotti* (Table 1) is crucial in guiding therapy. In this case, MALDI-TOF mass spectrometry [22,24] performed within 24 h of colony growth allowed rapid species-level confirmation and timely initiation of high-dose voriconazole [24]. Notwithstanding the isolate’s minimum inhibitory concentration of 0.5 µg/mL, the infection progressed to endophthalmitis despite aggressive dosing, highlighting that even isolates with in vitro susceptibility profiles deemed to be favourable as per prior studies [10] may fail clinically when posterior extension occurs. A critical corollary is that when disease progression is evident, clinicians should maintain a low threshold for early surgical intervention [25], such as therapeutic penetrating keratoplasty (TPK), as medical salvage rates decline sharply once the posterior segment is seeded.

Although direct evidence remains limited, novel antidiabetic therapies such as SGLT2 inhibitors and GLP-1 receptor agonists through improved metabolic control and modulation of host immune function could influence susceptibility to opportunistic ocular infections, including fungal keratitis. There is a significant scope for systematic investigation in future studies [29].

Distinguishing *P. lilacinum* from other *Purpureocillium* species early in the diagnostic workflow is critical for guiding therapy, anticipating potential treatment failure, and determining the optimal timing for surgical intervention [25]. In vitro MIC data alone may not predict clinical outcomes, particularly in high-risk patients or when posterior extension is imminent. The lack of established clinical breakpoints for *P. lilacinum* complicates interpretation of MIC data interpretation, knowledge of clinical context, site of infection, PK/PD of antifungal drugs and patient’s immune status are therefore critical for therapeutic decision making [10,24].

Epidemiologic observations further emphasize the need for vigilance. A 2024 outbreak in a New York City ophthalmology clinic, involving 23 cases of keratitis linked to contaminated instrumentation, demonstrates both the outbreak potential of this mold and the importance of immediate public health notification upon isolation [30]. In this cluster, as in the present case, soft contact lens wear and antecedent topical corticosteroid use were the most common predisposing factors. These findings highlight the necessity of rigorous patient education regarding contact lens hygiene and the routine referral of rare ocular mold isolates to reference laboratories, both for definitive species identification and contribution to regional surveillance networks capable of detecting clonal spread [30].

Finally, the case underscores the importance of a multidisciplinary approach [28]. Infectious disease consultation facilitated dose optimization and drug-level monitoring, while corneal and vitreoretinal surgeons provided real-time assessment of surgical thresholds [28]. Such coordinated care should be regarded as the standard for *P. lilacinum* ocular infection, given the narrow therapeutic window before irreversible structural and functional damage occurs.

This report reinforces *P. lilacinum* as a low-incidence yet high-consequence ocular pathogen. The severe clinical course observed in a diabetic contact lens wearer, culminating in enucleation despite rapid diagnosis and guideline-concordant therapy, highlights three actionable imperatives: maintain a high index of suspicion when risk factors converge, expedite species-level identification to guide triazole therapy, and adopt an early, assertive surgical strategy when MICs are elevated, or posterior extension is imminent. In fungal keratitis, time is vision and with *P. lilacinum*, delay may cost the eye itself.

## Figures and Tables

**Table 1:** Comparative features of *Purpureocillium lilacinum* and *Purpureocillium variotii*.

Feature	*P. lilacinum*	*P. variotii*
Epidemiology	Rare cause of human infection; disproportionately represented among severe filamentous keratitis and post-surgical ocular mold infections; increasingly reported in contact lens-associated keratitis [14].	Less frequently associated with ocular disease; more common in onychomycosis, cutaneous infections, and respiratory colonization; ocular involvement is rare [24].
Morphology (Culture)	Colonies typically fast-growing (2–3 days), lilac to violet with a velvety texture; conidiophores slender and branched, with chains of elliptical conidia [26].	Colonies pale to tan or yellow-brown in 2–3 days; conidiophores thicker, producing ellipsoidal to oblong conidia; morphology less distinctive in early growth [26].
Molecular Identification	ITS sequencing and MALDI-TOF MS can reliably distinguish from *P. variotii*; closely related to other *Purpureocillium* spp. [22,27].	Similarly identifiable via ITS sequencing and MALDI-TOF MS; often misidentified morphologically without molecular tools [22,27].
Antifungal Susceptibility	High MICs noted for amphotericin B, fluconazole, itraconazole and flucytosine. Echinocandins show poor or variable activity. Voriconazole and posaconazole display the most reliable in vitro efficacy, with posaconazole typically yielding the lowest MICs. However, in vitro susceptibility does not always correlate with clinical outcome, particularly in deep-seated ocular infections [14,21,22,12,28].	Typically broader susceptibility than *P. lilacinum*; generally responsive to amphotericin B, echinocandins, and triazoles (voriconazole, posaconazole, isavuconazole). Clinical outcomes more favorable with standard antifungal therapy [22,24].
Therapeutic Implications	Requires early species-level identification to guide therapy; triazoles are first-line, but treatment failure may occur even with deemed susceptible-range MICs [25].	Broader therapeutic options available; standard antifungal classes (including amphotericin B) may be effective.
Clinical Course in Ocular Disease	Often aggressive with rapid corneal invasion and potential posterior extension; higher likelihood of requiring surgical intervention (TPK, enucleation) [25].	Slower progression; less frequently associated with globe-sacrificing outcomes; better prognosis with early antifungal therapy [24].

## Data Availability

The original contributions presented in this study are included in the article. Further inquiries can be directed to the corresponding author.
